# Decreasing healthcare-associated infections (HAI) is an efficient method to decrease healthcare-associated Methicillin-resistant S.aureus (MRSA) infections Antimicrobial resistance data from the German national nosocomial surveillance system KISS

**DOI:** 10.1186/2047-2994-1-3

**Published:** 2012-01-26

**Authors:** Petra Gastmeier, Frank Schwab, Michael Behnke, Christine Geffers

**Affiliations:** 1 Institute of Hygiene and Environmental Medicine, Charité-Universitätsmedizin Berlin, Hindenburgdamm 27, 12203 Berlin, Germany

**Keywords:** Surveillance, MRSA, epidemiology, *Staphylococcus aureus*

## Abstract

**Background:**

By analysing the data of the intensive care unit (ICU) component of the German national nosocomial infection surveillance system (KISS) during the last ten years, we have observed a steady increase in the MRSA rates (proportions) from 2001 to 2005 and only a slight decrease from 2006 to 2010. The objective of this study was to investigate the development of the incidence density of nosocomial MRSA infections because this is the crucial outcome for patients.

**Findings:**

Data from 103 ICUs with ongoing participation during the observation period were included. The pooled incidence density of nosocomial MRSA infections decreased significantly from 0.37 per 1000 patient days in 2001 to 0.15 per 1000 patient days in 2010 (RR = 0.40; CI95 0.29-0.55). This decrease was proportional to the significant decrease of all HCAI during the same time period (RR = 0.61; CI95 0.58-0.65).

**Conclusions:**

The results underline the need to concentrate infection control activities on measures to control HCAI in general rather than focusing too much on specific MRSA prevention measures. MRSA rates (proportions) are not a very useful indicator of the situation.

## Findings

Methicillin resistant *S. aureus *(MRSA) is the major focus of public awareness of healthcare-associated infection (HCAI) problems in many countries and surveillance should support the work toward decreasing nosocomial MRSA infections. However, by analysing the data of the intensive care unit (ICU) component of the German national nosocomial infection surveillance system (KISS), we have observed a steady increase in the MRSA rate as a percentage of nosocomial MRSA infections among all nosocomial *S. aureus *infections from 2001 to 2005 and only a slight decrease in the period from 2006 to 2010. The objective of this study was to investigate the development of the incidence density of nosocomial MRSA infections during the observation period, because this is the crucial outcome for patients.

The ICU surveillance method used in KISS is almost identical to the method used by the National Healthcare Surveillance Network (NHSN) for surveillance of HCAI in ICUs [1], [2]. Participation in KISS is voluntary and results are handled confidentially. HCAI are mainly registered by infection control practitioners but also by physicians. They are trained in KISS methodology and in applying CDC definitions for HCAI. The number of participating ICUs has increased from year to year. Up to 2004, the ICUs received their data twice a year to provide feedback and encourage infection control activities; since 2005 they have had the opportunity to analyse their data whenever they want, due to the introduction of a web-based data management system. In the case of an HCAI, up to four pathogens probably responsible for the infection can be recorded. As well as their HCAI rates, the ICUs also receive a list with their case patients and the pathogens probably responsible.

The ICU KISS data from January 2001 to December 2010 that were used for this analysis focused on microbiologically-confirmed primary bloodstream infections, X-ray-confirmed nosocomial pneumonia and nosocomial urinary tract infections accordingly because the definitions for these infection types did not change during the observation period in KISS. Only those ICUs that provided data for at least 3 months per year during the entire study period were included. The MRSA rate (proportion of nosocomial MRSA/nosocomial *S. aureus *infections) and the incidence densities of nosocomial Methicillin susceptible *S. aureus *(MSSA) infections and nosocomial MRSA infections (both per 1000 patient days) were calculated. Relative risks (RR) with 95% confidence intervals were determined to compare the yearly pooled means of nosocomial MRSA infection incidence densities. In addition, the development of all HCAI in general was described by calculating RR and 95% confidence intervals to compare the yearly pooled means of all HCAI incidence densities.

**Figure 1:**
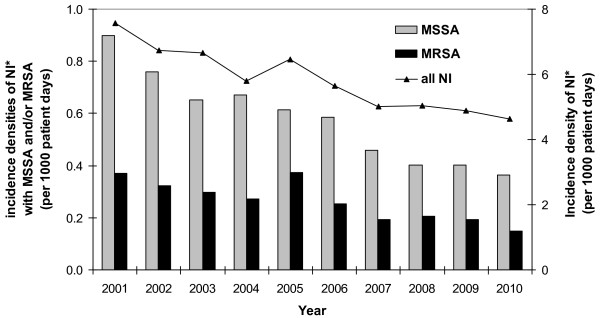
**Incidence densities of nosocomial infections with MRSA and MSSA and all nosocomial infections**. NI, nosocomial infections; MSSA, Methicillin sensitive *S. aureus*; MRSA, Methicillin resistant *S.aureus*, focused on microbiologically-confirmed primary bloodstream infections, X-ray-confirmed nosocomial pneumonia and nosocomial urinary tract infections.

Data from 103 ICUs, about 1 million patients with about 3.5 million patient days and a total of 20,504 HCAI were included. Among the HCAI were 2,026 nosocomial MSSA infections and 921 nosocomial MRSA infections (Table 1 and 2). The pooled mean MRSA rate (nosocomial MRSA infections/nosocomial *S. aureus *infections) increased from 2001 to 2005 and decreased slightly until 2010 compared to the starting point. However, the pooled incidence density of nosocomial MRSA infections decreased significantly from 0.37 per 1000 patient days in 2001 to 0.15 per 1000 patient days in 2010 (RR = 0.40; CI95 0.29-0.55) (Figure 1). This decrease was proportional to the significant decrease of all HCAI during the same time period (RR = 0.61; CI95 0.58-0.65) (Figure 1).

**Table 1:** Characteristics of 103 ICUs with continuous participation in ICU-KISS from 2001 to 2010.

Parameter	No. (IQR; %)
Analysed months per ICU, median	114 (IQR 108-119)

Type of ICU	

surgical	27 (26.2%)

surgical-medical	52 (50.5%)

medical	12 (11.7%)

other speciality	12 (11.7%)

Type of hospital	

university hospital ICUs	17 (16.5%)

teaching hospital ICUs	56 (54.4%)

non academic hospital ICUs	30 (29.1%)

Size of ICU (median number of beds)	11 (IQR 9-14)

Size of Hospital (median number of beds)	500 (IQR 315-1000)

ICU, intensive care unit; No., number; IQR, interquartile range.

**Table 2:** Development of ICU surveillance data in 103 ICUs with ongoing surveillance from 2001-2010

Year	Patient days	Ventila-tor utiliza-tion rate	Noso-comial MSSA infections*	Noso-comial MRSA infections*	MRSA rate (%)	Incidence density of nosocomial MSSA infections*	Incidence density of nosocomial MRSA infections*	RR incidence densities of nosocomial MRSA infections* compared to baseline year 2001 (95%CI)	Incidence density of nosocomial infections*	RR incidence densities of nosocomial infections* compared to baseline year 2001 (95%CI)
2001	322990	42.3	290	120	29.3	0.90	0.37	1 = ref	7.56	1 = ref

2002	332215	41.7	252	107	29.8	0.76	0.32	0.87 (0.67-1.13)	6.74	0.89 (0.84-0.94)

2003	338819	42.1	221	101	31.4	0.65	0.30	0.8 (0.62-1.05)	6.66	0.88 (0.83-0.93)

2004	343513	42.8	230	93	28.8	0.67	0.27	0.73 (0.56-0.96)	5.80	0.77 (0.072-0.81)

2005	360684	42.9	221	135	37.9	0.61	0.37	1.01 (0.79-1.29)	6.46	0.85 (0.81-0.90)

2006	353148	42.5	207	89	30.1	0.59	0.25	0.68 0.52-0.89)	5.65	0.75 (0.70-0.79)

2007	363476	42.4	167	70	29.5	0.46	0.19	0.52 (0.39-0.70)	5.02	0.66 (0.62-0.71)

2008	372463	43.1	150	77	33.9	0.40	0.21	0.56 (0.42-0.74)	5.04	0.67 (0.63-0.71)

2009	371865	42.9	150	72	32.4	0.40	0.19	0.52 (0.39-0.70)	4.89	0.65 (0.61-0.69)

2010	380173	44.1	138	57	29.2	0.36	0.15	0.40 (0.29-0.55)	4.63	0.61 (0.58-0.65)

total	3539346	42.7	2026	921	31.3	0.57	0.26		5.80	

MSSA, Methicillin susceptible *S.aureus*; MRSA, Methicillin resistant S.aureus,;*nosocomial infections focused on microbiologically-confirmed primary bloodstream infections, X-ray-confirmed nosocomial pneumonia and nosocomial urinary tract infections

However, despite a general trend of relative unchanged proportion of MRSA among nosocomial S.aureus infection during the last 10 years, the participating ICUs were able to significantly decrease the incidence of nosocomial MRSA and MSSA infections by the same proportion (60%) through surveillance of nosocomial infections, feedback and appropriate intervention measures, whereas the overall HCAI incidence was decreased only by 39%. This development in KISS ICUs is in accordance with the development of the incidence of nosocomial MRSA infections in other countries. For example, US intensive care units observed a 49.6% decrease in the MRSA central line associated bloodstream infection (BSI) incidence in the period from 1997 to 2007 [3] and French intensive care units in the Paris area achieved a 59% decrease inf the MRSA burden from 1993 to 2007 [4].However, it is necessary to interpret the data carefully, because there are indeed some limitations. Firstly, the sensitivity of diagnosing HCAI may have changed during the study period. A decreasing rate of microbiology reports would lead to a decreasing incidence density of nosocomial MRSA infections. However, the annual proportion of HCAI with microbiology reports was 90.8% in 2001 and 89.4% in 2010. Secondly, the severity of illness of ICU patients may have changed. If ventilator utilization rates are taken as a surrogate parameter, this is not the case, as these rates were 42.3% in 2001 and 42.7% in 2010. Thirdly, CDC definitions for BSI and pneumonia changed during the observation period. However, by focusing on microbiologically-confirmed BSI and X-ray confirmed pneumonia only, instead of including all BSI and pneumonia cases, this should not have had an influence.

This significant decrease of HCAI MRSA infections was masked in Germany because the public only looked at the MRSA rate and did not recognize the significant decrease in the incidence of MRSA infections, at least in the group of KISS ICUs. The concentration on the proportion of MRSA and other multidrug resistant pathogen instead of surveying the incidence of MDR pathogens may lead to less awareness of the need for the prevention of HCAI in general. Other nosocomial pathogens may lead to similar or even higher attributable morbidity and mortality. Our results therefore underline the importance of concentrating on measures to control HCAI in general, rather than focusing too much on specific MRSA prevention measures.

## Competing interests

The authors declare that they have no competing interests.

## Authors' contributions

PG and CG are responsible for the concept, design and implementation of the ICU-KISS surveillance module. MB supervised the web-based platform for the surveillance module and managed the data collection. FS performed the statistical analysis and interpreted the data. PG drafted the manuscript. CG, MB and FS critically revised the manuscript. All authors read and approved the final manuscript.
